# The Gut Microbiota Metabolite Butyrate Modulates Acute Stress-Induced Ferroptosis in the Prefrontal Cortex via the Gut–Brain Axis

**DOI:** 10.3390/ijms26041698

**Published:** 2025-02-17

**Authors:** Zhen Wang, Xiaoying Ma, Weibo Shi, Weihao Zhu, Xiaowei Feng, Hongjian Xin, Yifan Zhang, Bin Cong, Yingmin Li

**Affiliations:** Collaborative Innovation Center of Forensic Medical Molecular Identification, Hebei Key Laboratory of Forensic Medicine, Department of Forensic Medicine, Hebei Medical University, Shijiazhuang 050017, China; 22033100273@stu.hebmu.edu.cn (Z.W.); 22031100052@stu.hebmu.edu.cn (X.M.); shiweibo@hebmu.edu.cn (W.S.); 24031100106@stu.hebmu.edu.cn (X.F.); 22033100279@stu.hebmu.edu.cn (H.X.); 23033100279@hebmu.edu.cn (Y.Z.)

**Keywords:** acute stress, prefrontal cortex, ferroptosis, gut–brain axis, butyrate

## Abstract

Stress has been implicated in the onset of mental disorders such as depression, with the prefrontal cortex (PFC) playing a crucial role. However, the underlying mechanisms remain to be fully elucidated. Metabolites secreted by intestinal flora can enter the bloodstream and exert regulatory effects on the body. Consequently, this study aims to investigate the molecular mechanisms by which gut flora influences ferroptosis in PFC neurons, thereby affecting depression-like behavioral changes in mice subjected to acute stress. Initially, we established a mouse model of acute restraint stress (3-day duration) and verified that stress-induced ferroptosis of PFC neurons contributed to depression-like behavioral alterations in mice, as evidenced by morphological, behavioral, and molecular biology assessments. Subsequently, through fecal microbiota transplantation (FMT) experiments, we established a significant correlation between gut microbiota and ferroptosis of PFC neurons in acute stress-exposed mice. 16S rDNA sequencing identified butyric acid-producing bacteria, specifically *g_Butyricimonas* and its primary metabolite, butyric acid, as critical regulators of ferroptosis in PFC neurons in acutely stressed mice. Furthermore, the intervention of butyrate demonstrated its potential to ameliorate damage to the intestinal and blood–brain barriers in these mice. This intervention also mitigated depression-like behaviors induced by ferroptosis of PFC neurons by alleviating systemic inflammatory responses. The findings of this study indicate that acute stress-induced ferroptosis of PFC neurons plays a critical role in depression-like behavioral changes in mice. Additionally, the gut microbiota metabolite butyrate can modulate ferroptosis and depression-like behavioral changes through the gut–brain axis.

## 1. Introduction

Stress is a nonspecific adaptive reaction to endogenous or exogenous stimuli, maintaining organismal homeostasis [1]. When the stressor is excessively intense and rapid, classified as acute stress, the resultant stress response surpasses the body’s adaptive capacity, potentially inducing various physiological or psychological disorders [2]. In the event of significant psychological trauma, such as earthquakes, fires, or car accidents, individuals initially experience acute stress disorder (ASD). If ASD is not effectively managed, it can progress to secondary depressive episodes. According to the Global Burden of Disease Study, depression affects approximately 3–4% of the global population [3,4]. Individuals suffering from depression frequently necessitate prolonged medical and psychological interventions, placing a substantial strain on both familial units and national healthcare infrastructure [5]. Consequently, elucidating the mechanisms underlying acute stress-induced bodily harm and depression constitutes an urgent scientific challenge within the realm of clinical medicine.

The prefrontal cortex (PFC), located in the anterior region of the frontal lobe, is critical for governing complex cognitive processes, emotional regulation, and decision-making [6]. Impairment and dysfunction of the PFC are associated with the onset and progression of schizophrenia [7], PTSD [8], and depression [9]. Empirical evidence indicates that stress induces atrophy in PFC pyramidal neurons, a reduction in dendritic spines, and pathological alterations in iron metabolism [10]. Iron, a critical element for the brain, is involved in oxygen transport, DNA synthesis, and mitochondrial respiration and generates hydroxyl free radicals that cause oxidative damage to lipids, proteins, and carbohydrates upon disruption of its metabolism [11]. Ferroptosis, an iron-dependent form of programmed cell death characterized by unrestricted lipid peroxidation, is important in the study of diseases like cancer and depression. A limited number of studies have reported that stress may induce alterations in ferroptosis markers within the PFC of mice [12]. However, the use of ferroptosis inhibitors for confirmation has not been undertaken. The relationship between acute stress and ferroptosis in PFC neurons of mice remains ambiguous, necessitating further investigation into the underlying mechanisms.

The gut microbiota comprises a diverse array of microorganisms residing in the human gastrointestinal tract [13], and is critical for maintaining physiological homeostasis [14]. Dysbiosis has been implicated in a range of diseases, including inflammatory bowel disease [15], autism, and depression [16,17]. Stress can induce alterations in intestinal morphology and physiology in mice, alongside modifications in gut microbiota, ultimately impacting brain health [18,19]. The gut–brain axis represents a bidirectional communication network connecting the gut and brain [20] and plays an important role in modulating stress-related brain damage [21]. Furthermore, the intestinal microbiota is intricately associated with ferroptosis, influencing body iron absorption and systemic iron homeostasis regulation [22]. Modulating gut microbiota composition has been shown to mitigate brain ferroptosis induced by ischemia–reperfusion or ischemic stroke [23,24]. However, the involvement of gut microbiota in acute stress-induced ferroptosis in PFC neurons remains to be elucidated.

In this study, we established an acute restraint stress mouse model and employed the ferroptosis inhibitors Ferrostatin-1 (Fer-1) and Deferoxamine (DFO) to investigate ferroptosis occurrence in PFC neurons under acute stress conditions. Subsequently, we used fecal microbiota transplantation (FMT) to elucidate the role of gut microbiota in PFC neuronal ferroptosis under acute stress. Concurrently, 16S rDNA sequencing technology was used to identify key gut bacteria and metabolites implicated in this process. Following this, the identified key metabolites were administered to mice experiencing acute stress. We measured various parameters including intestinal barrier permeability, serum inflammatory factor levels, blood–brain barrier permeability, and PFC inflammatory factor expression to investigate underlying mechanisms of PFC neuronal ferroptosis in the context of acute stress. This study identifies novel therapeutic targets for the prevention and treatment of stress-related mental health disorders.

## 2. Results

### 2.1. Acute Stress-Induced Ferroptosis of PFC Neurons Contributes to Depression-like Behavioral Alterations in Mice

**Figure 1:**
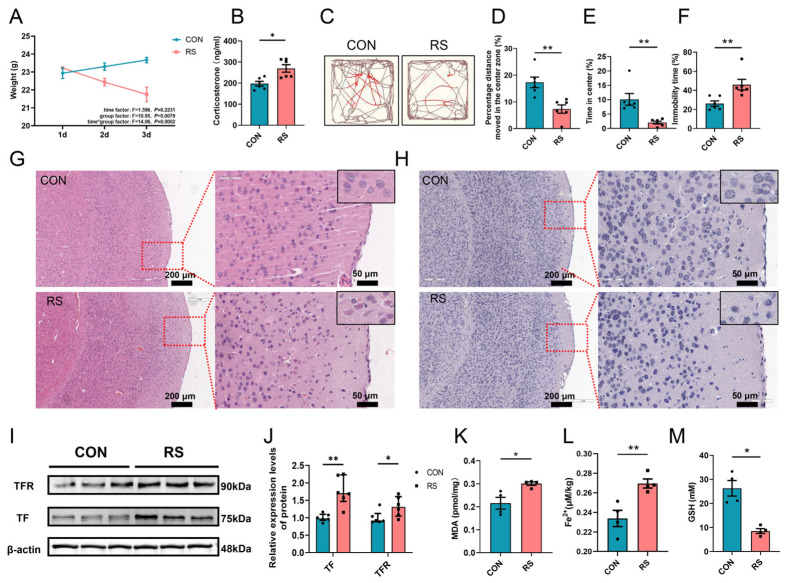
Acute stress induced depressive-like behavior and PFC neuronal damage in mice. (**A**) Body weight of the mice in the CON and RS groups over the initial three-day period. Data are expressed as mean ± SEM, with statistical significance assessed via two-factor repeated measures ANOVA. (**B**) Serum corticosterone levels in the two groups of mice. (**C**) Representative movement trajectories of the two groups of mice during the open field test. Red lines show the mice’s movement in the central zone, and brown lines indicate its trajectory in the peripheral zone. (**D**,**E**) In the open field test, the percentage of movement distance within the central area (**D**) and the percentage of residence time in the central area (**E**). (**F**) The percentage of immobile time in the tail suspension test. *n* = 6. (**G**,**H**) Representative images of HE staining (**G**) and Nissl staining (**H**) of the PFC in the two groups of mice. Scale bars: 200 μm and 50 μm. (**I**,**J**) The relative protein expression levels of TF and TFR in the PFC. *n* = 6. (**K**–**M**) The MDA content (**K**), Fe^2+^ content (**L**), and GSH content (**M**) in the PFC of the two groups of mice. *n* = 4. Data were presented as mean ± SEM. Statistical significance between the two groups was assessed using Student’s *t*-test for normally distributed data, and the Mann–Whitney rank sum test for data that did not follow a normal distribution. Significance levels were denoted as * *p* < 0.05 and ** *p* < 0.01.

Initially, we used an acute restraint stress murine model [25,26]. Our findings indicated a significant reduction in body weight over time in the restraint stress group (RS) compared to the control group (CON) (Figure 1A). Additionally, there was a marked increase in serum corticosterone levels in the RS mice (Figure 1B). Behavioral assessments revealed that the RS group exhibited depressive-like symptoms. Specifically, in the open field test; the RS mice demonstrated a significant decrease in both the percentage of distance traveled and the percentage of time spent in the central area (Figure 1C–E). In the tail suspension test, the RS group mice exhibited a significantly increased percentage of immobility time (Figure 1F). To investigate the underlying mechanisms of depression-like behavior in RS mice, we initially examined damage to PFC neurons across different groups using HE staining and Nissl staining. The HE staining results indicated that, in comparison to the CON group, the PFC of the RS group displayed edematous nerve cells, a higher number of red neurons with red cytoplasm, and a loss of clear nuclear structure (Figure 1G). Nissl staining results indicated that, in comparison to the CON group, the PFC neurons of the RS group exhibited mild edema, disappearance of Nissl bodies, cellular pyknosis, and increased staining density (Figure 1H). Furthermore, our analysis revealed alterations in markers associated with iron metabolism and lipid peroxidation in the PFC of RS mice. Specifically, compared to the CON group, the RS group showed significantly elevated expression levels of transferrin (TF) and transferrin receptor (TFR), as well as increased contents of Fe^2+^ and malondialdehyde (MDA), which are end products of lipid peroxidation. Conversely, the content of glutathione (GSH) was significantly reduced in the RS group (Figure 1I–M).

**Figure 2:**
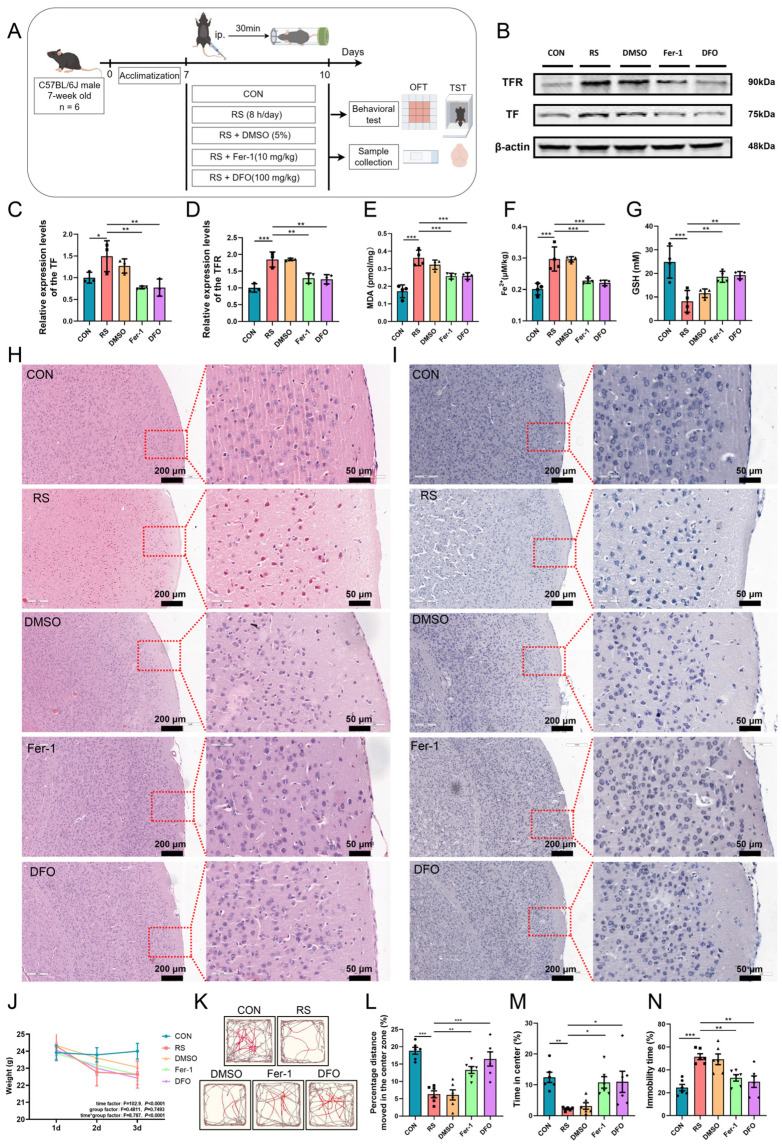
Acute stress-induced ferroptosis of PFC neurons is implicated in depression-like behavior in mice. (**A**) Schematic representation of the experimental protocol for administering the ferroptosis inhibitors. The figure was drawn by Figdraw, ID:RUUAO2fbf0. (**B**–**D**) Relative protein expression levels of TF (**C**) and TFR (**D**) in the PFC. *n* = 3. (**E**–**G**) MDA content (**E**), Fe^2+^ content (**F**), and GSH content (**G**) in the PFC of each group of mice. *n* = 4. (**H**,**I**) Representative histological images of HE staining (**H**) and Nissl staining (**I**) in the PFC of mice from the respective groups. Scale bars: 200 μm and 50 μm. (**J**) Body weight data of mice in each group were recorded over the first three days. The data are presented as mean ± SEM, and statistical significance was assessed using a two-factor repeated measures ANOVA. (**K**) Representative movement trajectories of each group of mice were captured during the open field test. Red lines show the mice’s movement in the central zone, and brown lines indicate its trajectory in the peripheral zone. (**L**,**M**) In the open field test, the percentage of movement distance in the central area (**L**) and the percentage of time spent in the central area (**M**). (**N**) The percentage of immobile time for each group of mice was determined during the tail suspension test. *n* = 6. Data are expressed as mean ± SEM. Statistical significance among multiple groups was assessed using one-way ANOVA. * *p* < 0.05, ** *p* < 0.01, *** *p* < 0.001.

Considering all the aforementioned indicators are associated with ferroptosis, we hypothesized that ferroptosis in PFC neurons contributes to acute stress-induced PFC injury and depression-like behavior in mice. To test this hypothesis, we administered ferroptosis inhibitors Fer-1 and DFO in an acute restraint stress mouse model. The experimental procedure is illustrated in Figure 2A. Our findings indicate that supplementation with Fer-1 and DFO significantly mitigated ferroptosis in the PFC of RS mice. Compared to the RS group, the expression levels of TF and TFR, as well as the contents of MDA and Fe^2+^, in the PFC of the Fer-1 and DFO groups were significantly reduced, while the content of GSH was significantly elevated (Figure 2B–G). Conversely, no significant changes were observed in these parameters within the DMSO group. These findings suggest that ferroptosis occurs in the PFC neurons of acutely stressed mice. Furthermore, we observed that the neuronal damage in the PFC was significantly ameliorated in the Fer-1 and DFO groups compared to the RS group (Figure 2H,I). Moreover, although there was no significant difference in body weight change between the Fer-1 and DFO groups (Figure 2J), the depression-like behavior of the mice was significantly alleviated (Figure 2K–N). These findings suggest acute stress-induced ferroptosis of PFC neurons is implicated in the manifestation of depression-like behaviors in mice.

### 2.2. Gut Microbiota Is Involved in Ferroptosis in the PFC of Acute Stress Mice

**Figure 3:**
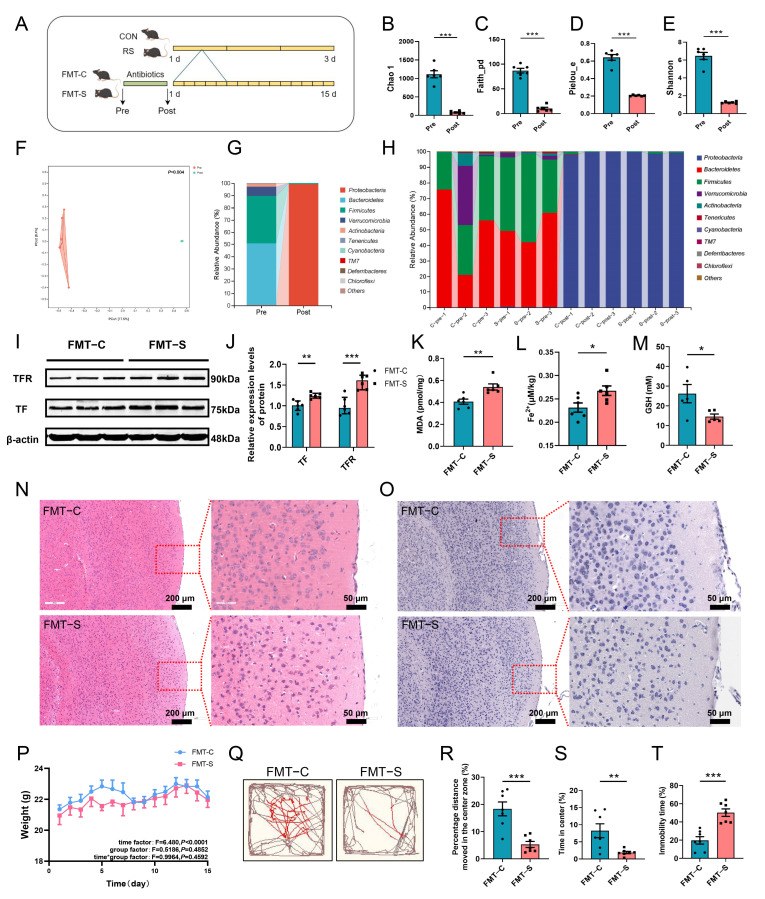
The gut microbiota is implicated in ferroptosis within the PFC of acutely stressed mice. (**A**) Experimental flowchart of FMT. The figure was drawn by Figdraw, ID:RWTIU78278. (**B**–**E**) Alpha diversity indices (Chao1, Faith’s_pd, Pielou_e, Shannon) of gut microbiota in mice before and after antibiotic treatment. (**F**) The beta diversity analysis of gut microbiota in mice before and after antibiotic treatment, utilizing PCoA analysis based on the Bray–Curtis distance. The Adonis test was employed to identify statistical differences between the two groups. (**G**,**H**) The relative abundance of gut microbiota at the phylum level before and after antibiotic treatment. *n* = 6. (**I**,**J**) The relative protein expression levels of TF and TFR in the PFC of mice from the FMT-C and FMT-S groups. (**K**–**M**) The MDA content ((**K**), *n* = 6), Fe^2+^ content ((**L**), *n* = 6), and GSH content ((**M**), *n* = 5) in the PFC of the two groups of mice. (**N**,**O**) Representative histological images of HE staining (**N**) and Nissl staining (**O**) in the PFC of mice from the FMT-C and FMT-S groups. Scale bars: 200 μm and 50 μm. (**P**) Monitoring of body weight changes in mice from the FMT-C and FMT-S groups over a 15-day period following fecal microbiota transplantation. Data are presented as mean ± SEM, and statistical significance was assessed using two-factor repeated measures ANOVA. (**Q**) Representative movement trajectories for each group of mice in the open field test. Red lines show the mice’s movement in the central zone, and brown lines indicate its trajectory in the peripheral zone. (**R**,**S**) In the open field test, the percentage of movement distance (**R**) and the percentage of residence time (**S**) in the central area. (**T**) The percentage of immobile time for each group of mice during the tail suspension test. *n* = 7. Data are expressed as mean ± SEM. The Student’s *t*-test was employed to assess the statistical significance between the two groups when the data followed a normal distribution. Conversely, the Mann–Whitney test was utilized to evaluate the statistical differences between the groups when the data deviated from a normal distribution. * *p* < 0.05, ** *p* < 0.01, *** *p* < 0.001. Pre: before antibiotic treatment. Post: after antibiotic treatment.

To investigate the involvement of gut microbiota in ferroptosis, we conducted FMT experiments, as illustrated in Figure 3A. Antibiotic-treated mice treated with an antibiotic mixture were utilized to ensure FMT efficacy. Sequencing analysis of the gut microbiota before and after antibiotic treatment demonstrated that the antibiotic regimen effectively eradicated most gut microbiota. Alpha diversity analysis revealed significant decreases in the Chao1, Faith_pd, Pielou_e, and Shannon indices in the Post mice (Figure 3B–E). Furthermore, gut microbiota structure analyses showed substantial differences between Pre and Post mice (Figure 3F). In the Pre mice, *p_Bacteroidetes*, *p_Firmicutes*, and *g_Lactobacillus* were predominant, whereas in the Post mice, *p_Proteobacteria* and *g_Enterobacter* emerged as the dominant intestinal bacteria (Figure 3G,H and Figure S1).

Following successful establishment of antibiotic-treated mice, fecal microbiota transplantation was conducted. Results indicated that compared to the FMT-C (feces from CON mice via gavage) group, the FMT-S (feces from RS mice via gavage) group exhibited significant upregulation of ferroptosis-related marker expression: TF and TFR (Figure 3I,J). Additionally, there was a notable increase in MDA and Fe^2+^ levels, along with a significant reduction in GSH content (Figure 3K–M). Furthermore, FMT-S mice demonstrated neuronal damage in the PFC analogous to RS mice (Figure 3N,O). However, no significant differences in body weight were detected between the FMT-S and FMT-C groups (Figure 3P). Nevertheless, FMT-S mice exhibited significant reductions in both moving distance and time spent in the central area during the open field test (Figure 3Q–S). Additionally, there was a notable increase in resting time observed in the tail suspension test (Figure 3T). These findings indicate that FMT effectively transferred the ferroptosis phenotype from RS mice to antibiotic-treated mice, resulting in prefrontal cortex neuronal damage and manifestation of depression-like behaviors in the antibiotic-treated mice.

### 2.3. Butyric Acid-Producing Bacteria Are the Key Enterobacteria Involved in Ferroptosis of PFC Neurons in Mice with Acute Stress

**Figure 4:**
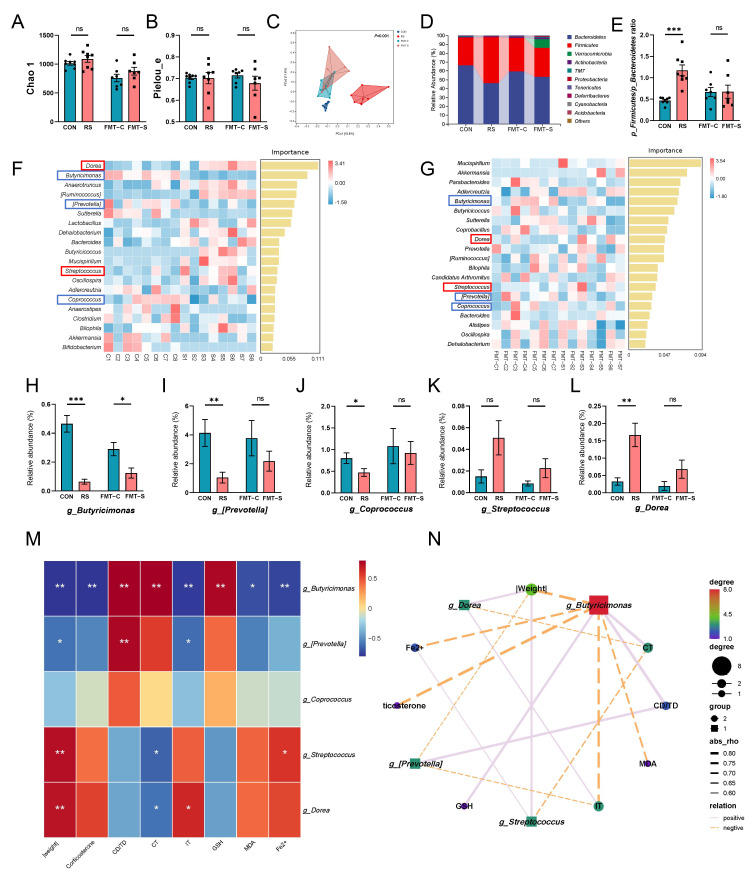
Butyric acid-producing bacteria are the primary enterobacteria implicated in the ferroptosis of PFC neurons in acutely stressed mice. (**A**,**B**) Alpha diversity indices of gut microbiota, including Chao1 (**A**) and Pielou_e (**B**). (**C**) Beta diversity analysis of gut microbiota across different groups of mice. (**D**) Relative abundance of gut microbiota at the phylum level in each group. (**E**) Ratio of the relative abundance of *p_Firmicutes* to *p_Bacteroidetes* in each group. CON, RS, *n* = 8; FMT-C, FMT-S, *n* = 7. (**F**) Random forest analysis was conducted to compare the gut microbiota between the mice in CON and RS groups. *n* = 8. (**G**) Random forest analysis was conducted to compare the gut microbiota between the mice in the FMT-C and FMT-S groups. *n* = 7. The key intestinal flora exhibiting consistent trends in both the RS and FMT-S groups are highlighted within a box. Blue indicates the intestinal flora that were reduced in the RS and FMT-S groups, while red indicates the intestinal flora that were increased in these groups. (**H**–**L**) The bar plots show the relative abundances of *g_Butyricimonas*, *g_[Prevotella]*, *g_Coprococcus*, *g_Dorea*, and *g_Streptococcus* in each group, based on the results of analyses (**F**) and (**G**). CON, RS, *n* = 8; FMT-C, FMT-S, *n* = 7. (**M**) Heat map illustrating the correlation analysis between key gut microbiota and indicators related to stress or ferroptosis. (**N**) Network diagram depicting the correlation analysis between key gut microbiota and indicators associated with stress or ferroptosis, *n* = 6. Data are presented as mean ± SEM. Statistical significance between the two groups was determined using Student’s *t*-test for normally distributed data, and the Mann–Whitney rank sum test for data that did not follow a normal distribution. * *p* < 0.05, ** *p* < 0.01, *** *p* < 0.001, ns = non significant. |Weight|: absolute value of the weight difference between the first day and last day treatment. CD/TD: percentage of distance moved in the central area of the open field experiment. CT: Percentage of time spent in the central area of the open field experiment. IT: Percentage of stationary immobility time in tail suspension experiments.

To investigate key gut bacteria involved in ferroptosis of PFC neurons in mice subjected to acute stress, we used 16S rDNA (V3–V4 region) sequencing technology to assess gut microbiota composition in mice undergoing the acute restraint stress and fecal microbiota transplantation. The findings indicated no significant difference in alpha diversity, as measured by the Chao1 and Pielou_e indices, between CON and RS mice (Figure 4A,B). However, significant β diversity differences were observed (Figure 4C). At the phylum level, both CON and RS intestinal microbiota were predominantly composed of *p_Bacteroidetes*, and *p_Firmicutes*. Relative to CON, RS had reduced *p_Bacteroidetes* and increased *p_Firmicutes,* resulting in a significantly elevated *p_Firmicutes*/*p_Bacteroidetes* ratio (Figure 4D,E). Furthermore, the gut microbiota composition of FMT-C and FMT-S mice showed significant differences, with dominant phyla reverting to *p_Bacteroidetes* and *p_Firmicutes*. However, no significant differences were observed in alpha diversity or *p_Firmicutes*/*p_Bacteroidetes* ratio between the two groups.

Random forest analysis was used to identify key intestinal bacteria associated with PFC ferroptosis in mice subjected to acute stress (Figure 4F,G). Comparative analysis between CON and FMT-C mice allowed us to focus on the intestinal flora exhibiting consistent changes in RS and FMT-S mice. The following genera were examined: *g_Butyricimonas, g_[Prevotella]*, *g_Coprococcus*, *g_Streptococcus*, and *g_Dorea*. All genera, except for *g_Streptococcus*, showed a close association with butyrate production, a key gut microbiota metabolite. The relative abundance of *g_Butyricimonas* [27], *g_[Prevotella]* [28], and *g_Coprococcus* [29], known butyric acid producers, was significantly reduced in RS mice intestines compared to CON mice. Conversely, *g_Dorea* [30], negatively correlated with butyric acid production, showed a significant increase. However, *g_Streptococcus*, unrelated to butyric acid production, tended to increase, although not significantly. Compared to FMT-C mice, FMT-S mice showed a similar trend in the aforementioned intestinal bacteria. Notably, the change in *g_Butyricimonas* was statistically significant (Figure 4H,L). Spearman correlation analysis results indicated that the abundance of *g_Butyricimonas*, *g_[Prevotella]*, and *g_Coprococcus* positively correlated with open field test central area moving distance and staying time, as well as GSH levels. Conversely, these genera negatively correlated with weight difference, corticosterone content, tail suspension test time percentage, and MDA and Fe^2+^ levels. In contrast, *g_Streptococcus* and *g_Dorea* showed an inverse relationship compared to *g_Butyricimonas*, *g_[Prevotella]*, and *g_Coprococcus*. Heatmap and network analyses results indicated that *g_Butyricimonas* is the primary gut microbiota associated with PFC ferroptosis (Figure 4M,N). These findings suggest that butyric acid-producing bacteria, exemplified by *g_Butyricimonas*, are significantly diminished in mice subjected to acute stress and play a crucial role in regulating PFC neuron ferroptosis under such conditions.

### 2.4. The Gut Microbiota Metabolite Butyrate Regulates Ferroptosis in PFC Neurons of Acutely Stressed Mice

**Figure 5:**
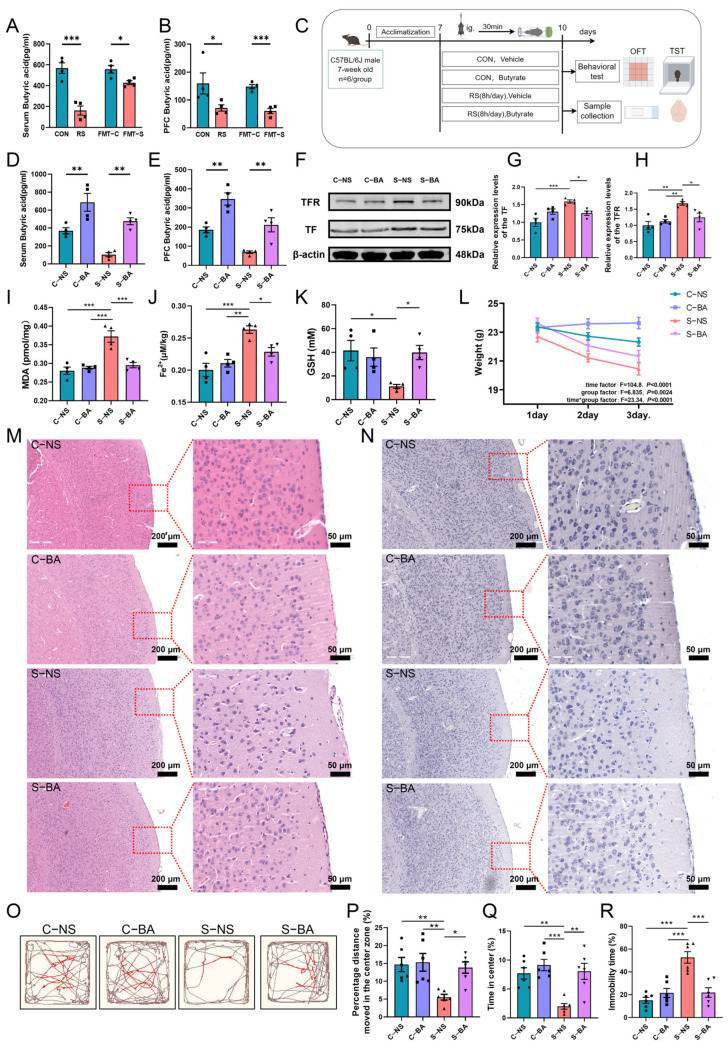
The gut microbiota metabolite butyrate modulates ferroptosis in PFC neurons of acutely stressed mice. (**A**,**B**) Quantification of butyric acid levels in serum and PFC tissues of mice across the CON, RS, FMT-C, and FMT-S groups. *n* = 4. (**C**) Schematic representation of the experimental design for the butyric acid treatment. The figure was drawn by Figdraw, ID:IRPWW4b47d. (**D**,**E**) Measurement of butyric acid concentrations in serum and PFC tissues of mice subjected to the butyrate treatment. (**F**–**H**) Analysis of relative protein expression levels of TF (**G**) and TFR (**H**) in the PFC across different groups. (**I**–**K**) The MDA content (**I**), Fe^2+^ content (**J**), and GSH content (**K**) in the PFC of each group of mice. *n* = 4. (**L**) Body weight measurements of mice subjected to the butyrate treatment across the first three days. *n* = 6. Data are expressed as mean ± SEM, with statistical significance assessed via two-factor repeated measures ANOVA. (**M**,**N**) Representative histological images of HE staining (**M**) and Nissl staining (**N**) of the PFC for each group of mice are provided. Scale bars: 200 μm and 50 μm. (**O**) Representative movement trajectories of each group of mice during the open field test. Red lines show the mice’s movement in the central zone, and brown lines indicate its trajectory in the peripheral zone. (**P**,**Q**) In the open field test, the percentage of movement distance within the central area (**P**) and the percentage of residence time in the central area (**Q**). (**R**) The percentage of immobile time in the tail suspension test. *n* = 6. Data are presented as mean ± SEM. Statistical significance across multiple groups was assessed using one-way ANOVA, with significance levels indicated as follows: * *p* < 0.05, ** *p* < 0.01, *** *p* < 0.001.

*g_Butyricimona* and other butyric acid-producing bacteria primarily synthesize butyric acid. Consequently, we initially quantified butyric acid levels in mouse serum and PFC for each experimental group. Findings indicated significantly lower butyric acid concentrations in RS and FMT-S group serum and PFC compared to the CON and FMT-C groups (Figure 5A,B). To investigate butyrate’s potential role in PFC neuron ferroptosis under acute stress, we conducted butyrate intervention experiments (Figure 5C). Butyrate supplementation markedly elevated serum and PFC butyric acid concentrations in the C-BA (butyrate) and S-BA (acute restraint stress + butyrate) groups compared to the C-NS (normal saline) and S-NS (acute restraint stress + normal saline) groups (Figure 5D,E). Subsequently, ferroptosis was assessed across all groups. Findings indicated relative to C-NS mice, S-NS mice exhibited significantly increased TF and TFR expression, elevated MDA and Fe^2+^ content, and reduced GSH levels in the PFC. Conversely, C-BA mice did not show significant alterations (Figure 5F–K). Compared with S-NS mice, the aforementioned ferroptosis indices in the PFC of S-BA mice exhibited significant improvement.

Histological examination using HE staining and Nissl staining revealed that, relative to the C-NS group, the C-BA group of mice did not display significant damage to the PFC. In contrast, the S-NS group of mice exhibited pathological alterations, including edema of certain neurons, an increased number of red neurons with red cytoplasm, loss of Nissl bodies, and cellular pyknosis and hyperchromasia (Figure 5M,N). Furthermore, in comparison to the C-NS group, the S-NS group exhibited a significant reduction in body weight (Figure 5L), the percentage of moving distance, and the percentage of time spent in the central area of the open field test (Figure 5O–Q). Additionally, the percentage of stationary time was markedly increased in the tail suspension test (Figure 5R). Notably, following butyrate supplementation, there was a significant amelioration in both the damage to PFC neurons and the depression-like behaviors in the S-BA group (Figure 5M–R). These findings indicate that butyrate has the potential to regulate ferroptosis in PFC neurons in mice subjected to acute stress. This regulation may consequently mitigate PFC neuronal damage and reduce depression-like behaviors in these mice.

### 2.5. Acute Stress Promotes Ferroptosis by Inducing Inflammation in the PFC

**Figure 6:**
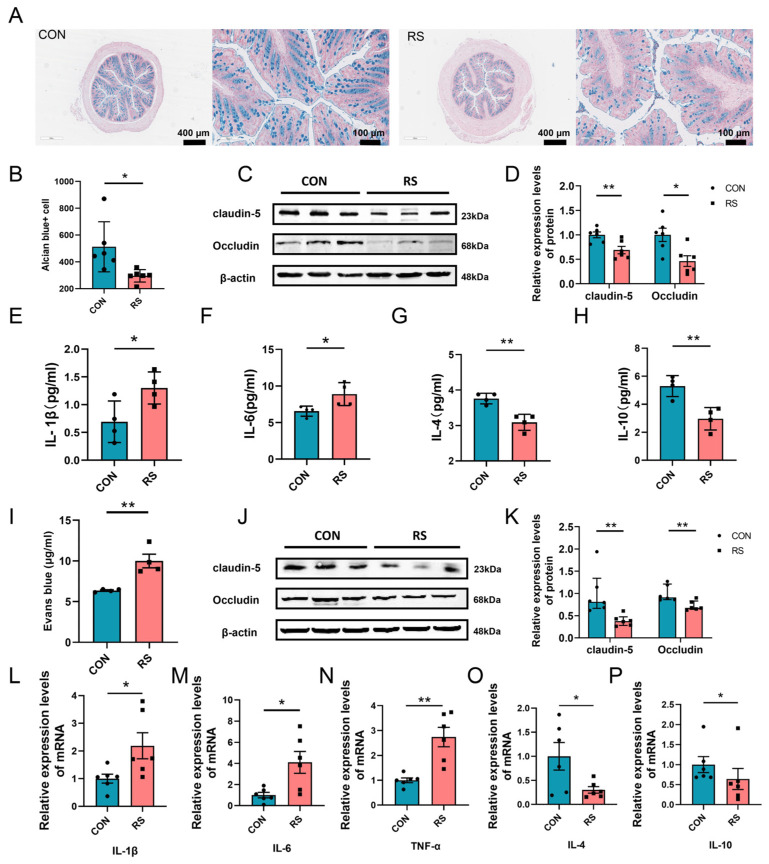
Acute stress promotes ferroptosis by inducing inflammation in the PFC. (**A**) Representative images of Alcian blue staining of colon tissues from mice in the CON group and RS group. Scale bars: 400 μm and 100 μm. (**B**) Quantification of Alcian blue-positive cells in colon tissues across the two groups. (**C**,**D**) Relative expression of tight junction proteins Claudin-5 and Occludin in colon tissues from the two groups. *n* = 6. (**E**–**H**) Levels of IL-1β (**E**), IL-6 (**F**), IL-4 (**G**), and IL-10 (**H**) in the serum of mice in the two groups. *n* = 4. (**I**) Quantification of Evans blue content in the PFC of CON and RS mice. *n* = 4. (**J**,**K**) Relative expression of tight junction proteins Claudin-5 and Occludin in the PFC from mice in the two groups. *n* = 6. (**L**–**P**) Assessment of relative mRNA expression of inflammatory cytokines IL-1β (**L**), IL-6 (**M**), TNF-α (**N**), IL-4 (**O**), and IL-10 (**P**) in the PFC of mice from the CON and RS groups. *n* = 6. Data are presented as mean ± SEM. The Student’s *t*-test was employed to assess statistical significance between the two groups when the data adhered to a normal distribution. Conversely, the Mann–Whitney rank sum test was utilized to evaluate statistical differences between the two groups when the data did not conform to a normal distribution. * *p* < 0.05, ** *p* < 0.01.

To further investigate the mechanisms by which key intestinal flora and their metabolites influence ferroptosis in PFC neurons during acute stress, we initially assessed intestinal barrier indices in mice from both the CON and RS groups. Alcian Blue Stain results demonstrated a significant reduction in the number of Alcian blue-positive cells in the colon tissue of the RS group compared to the CON group (Figure 6A,B). Furthermore, the expression levels of tight junction proteins Occludin and Claudin-5 were markedly decreased in the colon tissue of RS mice (Figure 6C,D), suggesting substantial impairment of the intestinal barrier in these mice. Disruption of the intestinal barrier can facilitate translocation of harmful substances from the intestine into the bloodstream, thereby triggering an inflammatory response [31]. Consequently, we measured serum levels of inflammatory markers. Results indicated that, in comparison to the CON group, the RS group exhibited a significant increase in serum levels of pro-inflammatory cytokines IL-1β and IL-6, alongside a significant decrease in serum levels of anti-inflammatory cytokines IL-4 and IL-10 (Figure 6E–H). Simultaneously, our findings revealed a significant increase in the content of Evans blue in the PFC of the RS group compared to the CON group. Additionally, there was a marked decrease in the expression of tight junction proteins Occludin and Claudin-5 in the PFC (Figure 6I–K). These results indicate a significant increase in blood–brain barrier permeability in the RS group mice. Compromised integrity of the blood–brain barrier can facilitate entry of inflammatory factors and other deleterious substances into the brain, thereby inducing neuroinflammation [32]. Subsequently, we investigated the inflammatory status of the PFC in CON and RS mice. Results indicated that the mRNA levels of pro-inflammatory cytokines IL-1β, IL-6, and TNF-α were significantly elevated in the PFC of the RS group compared to the CON group. Conversely, the mRNA levels of the anti-inflammatory cytokines IL-4 and IL-10 were significantly reduced in the RS group relative to the CON group (Figure 6L–P). These findings indicate that acute stress compromises the integrity of the gut barrier and blood–brain barrier, while simultaneously amplifying the systemic inflammatory response and the inflammatory state within the PFC. This may represent a pivotal mechanism through which key gut microbiota and their metabolites influence ferroptosis in PFC neurons in mice.

### 2.6. Butyrate Alleviates Ferroptosis of PFC Neurons in Acute Stress Mice by Regulating Inflammation Through Gut–Brain Axis

**Figure 7:**
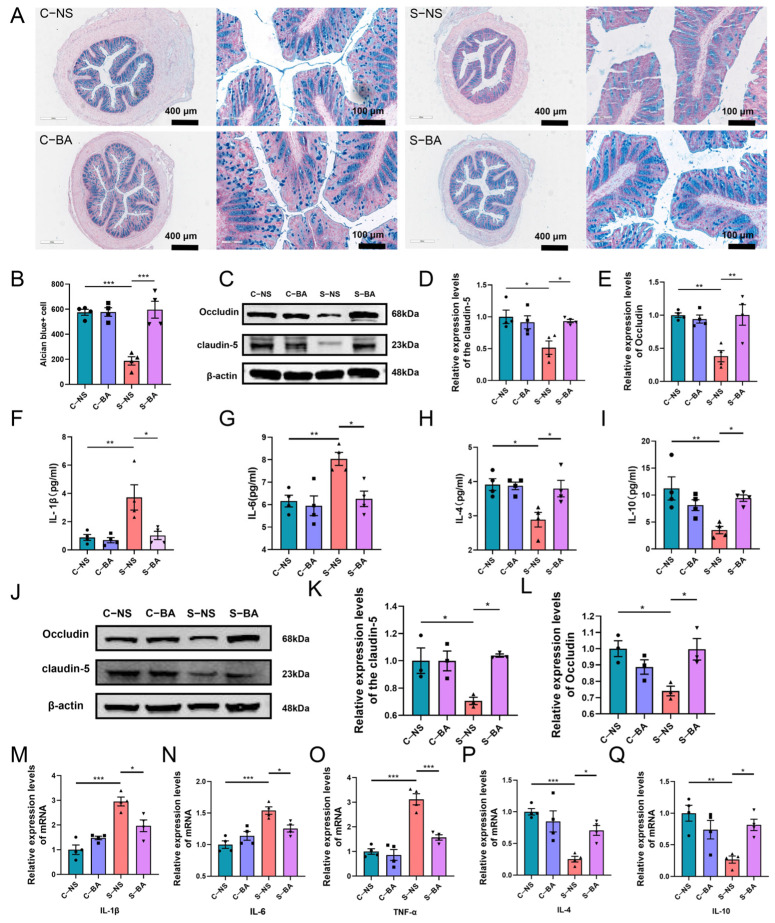
Butyrate mitigates ferroptosis in prefrontal cortex neurons of mice subjected to acute stress by modulating inflammation along the gut–brain axis. (**A**) Representative images of Alcian blue staining in colonic tissues from mice in the C-NS, C-BA, S-NS, and S-BA groups. Scale bars: 400 μm and 100 μm. (**B**) Quantification of Alcian blue-stained positive cells in colonic tissues across the four groups. (**C**–**E**) Relative expression of tight junction proteins Claudin-5 and Occludin in colonic tissues of the four groups. (**F**–**I**) Serum levels of IL-1β (**F**), IL-6 (**G**), IL-4 (**H**), and IL-10 (**I**) in the four groups. *n* = 4. (**J**–**L**) Relative expression of tight junction proteins Claudin-5 and Occludin in the PFC of the four groups. *n* = 3. (**M**–**Q**) Relative mRNA expression of inflammatory cytokines IL-1β (**M**), IL-6 (**N**), TNF-α (**O**), IL-4 (**P**), and IL-10 (**Q**) in the PFC of mice from the four groups. *n* = 4. Data are presented as means ± SEM. Statistical significance was determined using one-way ANOVA across multiple groups, with significance thresholds set at * *p* < 0.05, ** *p* < 0.01, *** *p* < 0.001.

To elucidate the mechanism by which butyrate mitigates ferroptosis in PFC neurons of acutely stressed mice, we investigated alterations in the gut barrier and blood–brain barrier, as well as inflammation levels in the serum and the PFC of RS mice following butyrate supplementation. The results indicated that, in comparison to the C-NS group, the S-NS group exhibited a significant reduction in the number of Alcian Blue-positive cells within colon tissue (Figure 7A,B). Additionally, there was a marked decrease in the expression levels of tight junction proteins Occludin and Claudin-5 (Figure 7C–E). Furthermore, serum analysis revealed a significant increase pro-inflammatory cytokines IL-1β and IL-6, alongside a significant decrease anti-inflammatory cytokines IL-4 and IL-10 (Figure 7F–I). Moreover, the expression levels of tight junction proteins Occludin and Claudin-5 in the PFC of the S-NS group were significantly reduced (Figure 7J–L). Concurrently, the mRNA levels of pro-inflammatory cytokines IL-1β, IL-6, and TNF-α in the PFC were markedly elevated, whereas the mRNA levels of anti-inflammatory cytokines IL-4 and IL-10 were significantly diminished (Figure 7M–Q). In comparison to the C-NS group, the C-BA group did not exhibit significant differences in the aforementioned indicators. However, in comparison to the S-NS group, the S-BA group supplemented with butyric acid exhibited significantly enhanced improvements in the aforementioned indicators. These findings suggest that butyrate supplementation can mitigate damage to the gut barrier and blood–brain barrier, attenuate the inflammatory response, and ameliorate ferroptosis in prefrontal cortex neurons in mice subjected to acute stress.

## 3. Discussion

Stress refers to the physiological, psychological, and behavioral adaptive responses that individuals experience in response to changes in their environment. The impact of stress on the central nervous system has been a focus of research both domestically and abroad. While moderate stress can provide protective benefits to the body, severe acute stress can directly disrupt central nervous system function. In this study, we investigated the role of gut microbiota in ferroptosis of PFC neurons in mice subjected to acute stress. We identified key gut bacteria and metabolites, laying the groundwork for further investigation into the mechanisms underlying acute stress-induced ferroptosis of PFC neurons.

Ferroptosis is characterized by peroxidation-induced destruction of phospholipid molecules with long chains of unsaturated fatty acids on cell or organelle membranes, ultimately leading to membrane rupture [33,34]. This cell death can be inhibited by the ferroptosis-specific inhibitor Fer-1 and the iron chelator DFO. Fer-1, an aromatic amine, scavenges lipid hydroperoxide radicals and reduces intracellular labile iron, exerting an anti-ferroptotic effect [35]. DFO, an extracellular iron chelator with antioxidant properties [36,37], mitigates ferroptosis. In this study, we aimed to elucidate ferroptosis in the PFC of mice subjected to acute stress. Fer-1 and DFO were administered post-restraint stress. Fer-1 and DFO treatment significantly attenuated expression of ferroptosis-related proteins TF and TFR, as well as Fe^2+^ accumulation in the PFC. Additionally, these treatments ameliorated the redox imbalance within the PFC and mitigated both PFC injury and depression-like behaviors in the acutely stressed mice. Previous research demonstrates brain iron metabolism homeostasis is primarily regulated by iron metabolism-related proteins, such as TF and TFR. Balanced iron ion levels in the brain depend on normal protein expression and cooperation [38,39]. When brain iron metabolism is dysregulated, there is increased free iron within the brain; elevated Fe^2+^ can initiate the Fenton reaction, generating reactive free radicals that disrupt intracellular redox homeostasis [40]. Furthermore, GSH serves as the primary intracellular scavenger of free radicals [41]. Reduced GSH levels precipitate lipid peroxidation inducing ferroptosis. De Sousa et al. have demonstrated that chronic unpredictable stress induces abnormalities in the levels of GSH and MDA within the PFC. Similarly, Wang et al. reported that psychological stress results in an elevated Fe^2+^ content in the PFC [42,43]. However, neither investigation employed ferroptosis inhibitors to explicitly define ferroptosis. Our study utilized ferroptosis inhibitors Fer-1 and DFO to demonstrate acute stress induces ferroptosis in PFC neurons, contributing to depression-like behaviors.

The gut microbiota represents the body’s most extensive and critical microecosystem, playing a pivotal role in the onset and progression of numerous diseases [44]. Research has demonstrated a close association between intestinal flora and stress, with various stressors markedly influencing microbial diversity within the gut [45]. For instance, stress has been shown to induce significant alterations in the abundance of *g_Lactobacillus* and *g_Bifidobacterium* in the intestines of mice [46,47]. Furthermore, gut microbiota have been implicated in the regulation of ferroptosis. The intestinal flora are crucial in iron metabolism, as they can induce the expression of hepcidin and contribute to the maintenance of systemic iron homeostasis [48,49]. Empirical evidence suggests that dysbiosis of the intestinal flora can trigger ferroptosis within the gastrointestinal tract. Conversely, the administration of probiotics has been shown to mitigate iron overload and lipid peroxidation, thereby inhibiting ferroptosis [50]. Therefore, we hypothesize that gut microbiota may play a role in ferroptosis within the PFC of mice subjected to acute stress, subsequently leading to depression-like behaviors. To evaluate this hypothesis, we conducted FMT experiments in mice. FMT involves the transfer of intestinal microbiota from a donor to a recipient and is utilized to investigate the causal relationship between intestinal flora and various health conditions or diseases [51]. Research has demonstrated that transplantation of feces from mice with stress-induced depression into SPF mice can induce intestinal inflammation and depressive phenotypes [52]. In the present study, we transplanted fecal microbiota from both the CON group and the RS group into antibiotic-treated mice. Our findings revealed that fecal microbiota transplantation effectively transferred the ferroptosis phenotype and depression-like behaviors observed in RS mice to the antibiotic-treated mice, demonstrating the gut microbiota’s involvement in PFC ferroptosis following acute stress. Interestingly, FMT showed no significant effect on body weight, potentially due to an insufficient duration for microbiota-mediated weight regulation or a limited functional capacity of donor microbiota to induce weight changes.

Based on these findings, we subsequently analyzed gut microbiota composition in mice subjected to acute stress and FMT. *g_Butyricimonas*, *g_[Prevotella]*, *g_Coprococcus*, and *g_Dorea*, implicated in butyric acid production, exhibited similar trends in both RS and FMT-S mice when compared to CON and FMT-C mice. Previous research has identified *g_Butyricimonas*, *g_[Prevotella]*, and *g_Coprococcus* as primary butyric acid producers [27,28,29], while a high abundance of *g_Dorea* is associated with reduced short-chain fatty acid (SCFA) producers [30]. Furthermore, our findings indicated that gut microbiota *g_Butyricimonas*, markedly diminished in both RS and FMT-S mice, was strongly associated with PFC damage and ferroptosis. The metabolite butyric acid was significantly reduced in the serum and PFC of these mice. Previous studies have demonstrated that *g_Butyricimonas* crucially maintains intestinal health via butyric acid production [53] and negatively correlates with proinflammatory responses [54]. We hypothesize diminished butyric acid-producing bacteria, specifically *g_Butyricimonas*, along with their metabolite butyrate, may critically involve PFC neuron ferroptosis in acutely stressed mice.

SCFAs, a group of organic fatty acids with one to six carbon atoms, including acetic acid, propionic acid, and butyric acid, play crucial roles in maintaining gut health, regulating immune function, modulating metabolism, and influencing nervous system function []. In this study, we focus specifically on butyric acid [55]. Butyric acid is critical for maintaining intestinal homeostasis and providing energy for colonocytes [56]. Previous research has demonstrated that butyrate can prevent pathogenic anemia through regulation of iron metabolism [57] and mitigate hepatocyte steatosis by decreasing lipid peroxidation [58], suggesting a role in ferroptosis onset and progression. Here, we investigated whether butyrate supplementation could alleviate PFC ferroptosis in a mouse model of acute stress. Results showed reduced PFC neuronal ferroptosis and improved depression-like behaviors in mice subjected to acute stress following butyrate supplementation, elucidating a protective role of the gut microbiota metabolite butyrate against PFC neuronal ferroptosis under acute stress conditions. Consistent with our findings, Chen et al. demonstrated butyrate can modulate ferroptosis, specifically noting butyrate alleviated ferroptosis in mouse colons with ulcerative colitis via Nrf2/GPX4 signaling pathway regulation [59]. Furthermore, Yang et al. reported transplantation of butyric acid-rich feces mitigated liver ferroptosis in mice with acute liver injury [60].

Notably, a bidirectional communication network, referred to as the “gut–brain axis”, exists between the gastrointestinal system and the brain [61,62,63]. We hypothesized that butyrate could influence ferroptosis in PFC neurons via the gut–brain axis in mice subjected to acute stress. Previous studies have demonstrated that imbalanced intestinal flora can compromise intestinal barrier integrity, reducing the expression of tight junction proteins [64]. Furthermore, compromised intestinal barrier integrity permits harmful substance translocation from the intestine into the bloodstream, eliciting chronic low-grade inflammation. This inflammatory state impairs the blood–brain barrier’s capacity to exclude deleterious agents, precipitating neuroinflammation [31,65]. Such aberrant inflammatory responses can disrupt iron metabolism and redox homeostasis, culminating in ferroptosis after inflammation activation and the associated signaling pathway [66]. Proinflammatory cytokines, such as IL-1β and IL-6, regulate ferritin synthesis, influencing iron storage within cells and tissues [67]. Sheng et al. demonstrated IL-6 can induce lipid peroxidation and iron imbalance in degenerative chondrocytes via the IL-6/miR–10a-5p/IL-6R axis, promoting ferroptosis [68]. Butyrate is an anti-inflammatory agent mitigating systemic inflammatory responses and reducing neuroinflammation levels [69,70,71]. These findings align with our study results wherein butyrate supplementation significantly mitigated damage to the intestinal and blood–brain barriers, reduced inflammation in both the serum and PFC, and improved ferroptosis in PFC neurons in acutely stressed mice.

The primary advantage of this study lies in the identification of ferroptosis in PFC neurons of mice subjected to acute stress, achieved through the application of ferroptosis inhibitors, and the elucidation of the role of butyric acid in this process. Nonetheless, the study has certain limitations. Firstly, in this study, male mice were selected to control for hormonal fluctuations and enable comparison with previous research findings. However, given that female mice are more susceptible to stress and exhibit higher depression rates, future studies will include female subjects to provide a more comprehensive understanding of stress-induced physiological damage and depression mechanisms. Secondly, while we have conducted a preliminary investigation into the inflammatory pathway as a mechanism by which butyrate influences ferroptosis, the intricate and multifaceted nature of the disease mechanisms necessitate further in-depth exploration to fully understand the underlying processes. For the key enterobacterium identified in our study, *g_Butyricimonas*, further investigation is required to determine its potential role in directly regulating ferroptosis and to elucidate the underlying mechanisms through single bacterial transplantation. Future research will concentrate on the impact of individual or multiple bacterial species on PFC neuronal damage and ferroptosis in stress-exposed mice. This line of inquiry may hold significant translational potential for the prevention and treatment of stress-related brain damage.

## 4. Materials and Methods

### 4.1. Animals

Male SPF C57BL/6J mice, aged 6–7 weeks and weighing 20–23 g, were obtained from the Beijing Weitong Lihua Laboratory Animal Center. The mice were housed in a temperature-controlled environment (20–22 °C) with 45 ± 5% humidity, provided with uniform sterile food and water, and maintained under a 12 h light/12 h dark cycle. Prior to the experiment, mice were acclimated to the new environment for 7 days. The study was conducted in strict accordance with the guiding principles of the Laboratory Animal Committee of Hebei Medical University. All experimental procedures were conducted in strict compliance with the Experimental Animal Research Protocol, as approved by the Experimental Animal Management Committee (IACUC-Hebmu-2023011, approval on 23 April 2023).

### 4.2. Experimental Design

#### 4.2.1. Acute Restraint Stress Model

A cylindrical polycarbonate tube, 12 cm in length and 2.4 cm in diameter, was constructed with air holes on both sides and equipped with movable valves at both ends. Mice assigned to the acute restraint stress group (RS, *n* = 6) were immobilized within the tube, and the valves at both ends were secured to prevent free movement or turning freely inside the polycarbonate (PC) tube [72]. The restraint procedure was performed for 8 h daily over 3 consecutive days. Mice in the control group (CON, *n* = 6) underwent daily fasting and water deprivation at consistent times, while being allowed normal feeding during the remaining periods [73].

#### 4.2.2. Ferroptosis Inhibitor Model

The acute restraint stress model was used, in which mice were administered ferroptosis inhibitors Fer-1 (17729-10, Cayman Chemical, Ann Arbor, MI, USA) and DFO (14595-1, Cayman Chemical, Ann Arbor, MI, USA). To prepare the solutions, the co-solvent dimethyl sulfoxide (DMSO) was utilized to dissolve Fer-1 and DFO to concentrations of 10 mg/mL and 100 mg/mL, respectively, in a 5% DMSO solution. The solutions were then diluted with sterile normal saline to final concentrations of 0.5 mg/mL and 5 mg/mL for intraperitoneal injection, administered 30 min prior to the restraint procedure. The experiment comprised five groups, each with six mice, as follows: the CON group (normal saline), the RS group (normal saline), the DMSO group (RS + 5% DMSO, 0.2 mL), the Fer-1 group (RS + Fer-1, 10 mg/kg) [74], and the DFO group (RS + DFO, 100 mg/kg) [75].

#### 4.2.3. Fecal Microbiota Transplantation

Fourteen male C57BL/6J mice, aged 5–6 weeks, were purchased from the Beijing Weitong Lihua Laboratory Animal Center for fecal microbiota transplantation. The mice were divided into two groups: FMT-C (feces from 4.2.1 CON mice via gavage) and FMT-S (feces from 4.2.1 RS mice via gavage), with seven mice in each group. All mice were given a combination of the antibiotics ampicillin (180 mg/kg/day), vancomycin (72 mg/kg/day), metronidazole (90 mg/kg/day), and imipenem (90 mg/kg/day) for three days prior to FMT to disrupt the intestinal microbiota [76]. Fresh antibiotic solutions were prepared daily to maintain efficacy, and antibiotics were purchased from Solarbio (Beijing, China). Fecal microbiota transfer was performed on day 4, ensuring a minimum 24-h post-antibiotic interval. Fresh fecal samples were collected daily from CON and RS mice and stored at −80 °C. Fresh feces were homogenized in sterile saline, centrifuged and the supernatant was administered to recipient mice via gavage. The FMT procedure was performed daily for 15 days [77].

#### 4.2.4. Butyrate Treatment Experiment

To investigate the impact of butyric acid on PFC ferroptosis in mice subjected to acute stress, the mice were divided into four groups (*n* = 6): C + NS (normal saline), C + BA (butyrate), S + NS (acute binding stress + normal saline), and S + BA (acute binding stress + butyrate). Sodium butyrate (1200 mg/kg; 303410-5G, Sigma-Aldrich, St. Louis, MO, USA) was administered by gavage to the mice 30 min prior to restraint for a duration of 3 days [78].

### 4.3. Feces Collection and 16S rDNA Sequencing

Fecal matter was collected from mice in each experimental group on the third day following the establishment of the acute restraint stress model, both prior to and subsequent to antibiotic treatment, as well as after fecal microbiota transplantation. Samples were stored in liquid nitrogen and then at −80 °C in a freezer.

Fecal samples were retrieved from the −80 °C freezer for DNA extraction using a DNA extraction kit (Omega Bio-Tek, Norcross, GA, USA). The concentration and purity of the extracted DNA were assessed using a NanoDrop NC2000 spectrophotometer (Thermo Fisher Scientific, Waltham, MA, USA) and agarose gel electrophoresis, respectively. The V3–V4 hypervariable region of the 16S rDNA was selected for polymerase chain reaction (PCR) amplification. The forward primer sequence was 5′-ACTCCTACGGGAGGCAGCA-3′, and the reverse primer sequence was 5′-GGACTACHVGGGTWTCTAAT-3′. PCR conditions were 25 cycles of denaturation at 98 °C for 30 s, annealing at 53 °C for 30 s, and extension at 72 °C for 45 s, followed by a final extension at 72 °C for 5 min. Post-PCR, amplicons were purified, quantified, pooled in equimolar amounts, and subjected to paired-end sequencing (2 × 250 bp) using the Illumina NovaSeq platform at Shanghai Personal Biotechnology Co., Ltd. (Shanghai, China). QIIME 2 version 2019.4 was utilized for microbiome bioinformatics research, and the DADA2 algorithm was employed to identify amplified sequence variants (ASVs) [79]. These were then classified and annotated using the Greengenes database [80,81].

### 4.4. Behavioral Assessment

#### 4.4.1. Open Field Test

The Open Field Test primarily assess depressive behavior, exploratory behavior, and locomotor activity in mice. The experimental setup included an open field reaction box and an automated data acquisition and processing system. The open field apparatus measured 25–30 cm in height and 72 cm in length. Prior to experimentation, mice were acclimated in a quiet laboratory environment for one hour. Each mouse was then placed at the center of the open field, and simultaneous video recording and timing were initiated. The movement trajectories of the mice were continuously recorded throughout the entire 5-min experiment. Upon completion, the video recording was terminated, and the percentage of total distance traveled within the central area and time spent in the central area were analyzed and recorded. Upon completion of each trial, the mouse’s excrement was removed, and the interior of the open field apparatus was sanitized with 75% ethanol. The ethanol was allowed to evaporate before subsequent experiments were conducted.

#### 4.4.2. Tail Suspension Test

The tail suspension test was used to assess depression-like behaviors in mice. Prior to experimentation, mice were acclimated to the laboratory environment for 1–2 h. Each mouse was carefully extracted from its cage, and its tail was promptly secured to minimize stress and anxiety. Medical tape was affixed 1 cm from the distal end of the mouse’s tail, enabling suspension from the device’s hanging stem. This configuration inverted the mouse head-down, with the tip of the tail 30 cm above the ground. The camera was horizontally aligned to comprehensively record the mouse’s behavior. The protocol required a 6-min video recording, with the final 5 min quantifying the animal’s immobilization duration. Upon test completion, the collection box was promptly cleaned of feces and urine.

### 4.5. HE Staining and Special Staining

The mice were anesthetized via intraperitoneal injection of 2% Pentobarbital (0.1 mL/20 g). Brain and colon tissues were excised, fixed in 10% neutral formalin, dehydrated through a graded series of alcohol concentrations, cleared in xylene, embedded in paraffin, oven-dried at 60 °C, and stored at 4 °C for future analysis. Prior to HE staining, the tissue sections underwent deparaffinization and hydration. This was followed by hematoxylin staining for 3 s, differentiation using hydrochloric acid alcohol, and eosin staining for 3 s. The tissue sections were then rinsed with tap water, dehydrated, cleared, and mounted. For toluidine blue and Alcian blue staining, we utilized the Nissl Stain Solution (Toluidine Blue Method) kit (G1436, Solarbio, Beijing, China) and the Alcian Blue Stain kit (G1560, Solarbio, Beijing, China), following the manufacturers’ protocols strictly. Observations and photographic documentation were conducted using an Olympus DP80 microscope (Olympus, Tokyo, Japan). ImageJ software (version 1.50b, National Institutes of Health, Bethesda, MD, USA) was employed for the quantification and analysis of micrograph data.

### 4.6. Western Blotting

The prefrontal cortex and colon tissues were homogenized in an ice bath using high-potency RIPA tissue lysate (R0010, Solarbio, Beijing, China) supplemented with the protease inhibitor PMSF. The homogenates were centrifuged at 12,000 rpm for 10 min at 4 °C to obtain clear lysates. Protein quantification was conducted using the bicinchoninic acid (BCA) Protein Assay kit (PC0020, Solarbio, Beijing, China). Equal amounts of proteins were separated by SDS-PAGE, transferred to PVDF membranes, and blocked using Y-Tec 5 min Ready-to-Use Blocking Buffer (YWB0501, Yoche, Shanghai, China). The membranes were incubated with primary antibodies: Transferrin (TF) (1:1000, cat: A1448, lot:5500004192, ABclonal, Wuhan, China), Transferrin receptor (TFR) (1:1000, cat: 13-6800, lot: RB5216, Invitrogen, Carlsbad, CA, USA), Claudin-5 (1:2000, cat: AF5216, lot: 23o5501, Affinity Biosciences, Jiangsu, China), Occludin (1:2000, cat: ET1701-76, lot: HM0527, Huabio, Hangzhou, China), and β-actin (1:200, cat: 66009-1-Ig, lot: 10044161, Proteintech, Wuhan, China). Secondary antibodies were goat anti-rabbit IgG (1:2000, cat: A23920, lot: ATVJ20071, Abbkine, Beijing, China) and goat anti-mouse IgG (1:2000, cat: A23710, lot: ATVG19051, Abbkine, Beijing, China). Membrane imaging was performed using the Odyssey imaging system (Odyssey V3.0, LI-COR Biosciences, Lincoln, NE, USA), and data analysis and quantification used ImageJ software.

### 4.7. Real-Time Quantitative PCR (RT-qPCR)

Total RNA was isolated from the prefrontal cortex tissue using TRIzol (15596018CN, Thermo Fisher Scientific, Waltham, MA, USA) and reverse transcribed into complementary DNA (cDNA) with a cDNA synthesis kit (A2791, Promega, Madison, WI, USA). Real-time quantitative PCR was then performed with a RT-qPCR kit (A6001, Promega, Madison, WI, USA). Each RT-qPCR reaction was run in triplicate, with GAPDH serving as the reference gene. The RT-qPCR data were normalized appropriately. The primer sequences used for RT-qPCR are detailed in Table 1.

### 4.8. Enzyme-Linked Immunosorbent Assay (ELISA)

Corticosterone (E-OSEL-M0001, Elabscience, Wuhan, China), IL-1β (SEA563Mu, Cloud-clone, Wuhan, China), and IL-6 (SEA079Mu, Cloud-clone, Wuhan, China) levels in mouse serum were quantified using commercial ELISA kits. Additionally, IL-4 (SEA077Mu, Cloud-clone, Wuhan, China), IL-10 (SEA056Mu, Cloud-clone, Wuhan, China), and butyric acid (CEO777Ge, Cloud-clone, Wuhan, China) levels in serum and PFC were measured. All procedures were performed in accordance with the manufacturers’ instructions.

### 4.9. Assessment of GSH, MDA, and Fe^2+^

The levels of glutathione (GSH), malondialdehyde (MDA), and Fe^2+^ were assessed using the GSH and oxidized glutathione (GSSG) Detection Kit (S0053, Biyantian, Shanghai, China), the Lipid Peroxidation MDA Assay Kit (S0131S, Biyantian, Shanghai, China), and the Tissue Iron Content Assay Kit (AKIC001M, Boxbio, Beijing, China), respectively, following the protocols provided by the manufacturers.

### 4.10. Evans Blue Assay

On the third day of confinement, mice were administered a 0.5% Evans Blue stain solution at a dosage of 8 mL/kg via the tail vein (G1810, Solebau, Beijing, China) [82]. The mice were anesthetized using 2% sodium pentobarbital (i.p., 40 mg/kg) 1 h after Evans blue dye injection and euthanized by intracardiac perfusion with saline. Brain tissue was then isolated to evaluate vascular leakage of the dye into the brain parenchyma. Half of the brain tissue was weighed and placed in a 1.5-mL centrifuge tube, to which 1 mL of 50% trichloroacetic acid diluted in 1× PBS was added. The tissue was rapidly homogenized and centrifuged at 10,000× *g* for 20 min. The OD value at 620 nm of the supernatant was measured using a spectrophotometer, and the OD values of standard Evans blue solutions with varying concentrations were determined for comparison. The standard curve was constructed, and the Evans blue content per unit weight of the tested sample was quantified based on this curve.

### 4.11. Statistical Analysis

Statistical analyses were conducted using GraphPad Prism 8 software (version 8.0.2, GraphPad Software, Santiago, Chile). Measurement data were presented as mean ± standard error of the mean (SEM). For normally distributed data, the Student’s *t*-test was employed to assess statistical differences between two groups, while one-way ANOVA was utilized to evaluate statistical significance across multiple groups. The Mann–Whitney test was used to assess statistical differences between two groups when the data did not conform to a normal distribution. For evaluating statistical significance among multiple groups, the Kruskal–Wallis test was employed. Additionally, repeated measures two-way ANOVA was conducted to determine the statistical significance of body weight variations across different groups. In addition, the Adonis test was used to assess β-diversity of gut microbiota, while Spearman correlation analysis evaluated the relationship between gut microbiota and biochemical indicators. A *p*-value of less than 0.05 was considered statistically significant.

## 5. Conclusions

In conclusion, our findings indicate that acute stress-induced ferroptosis of PFC neurons is implicated in depression-like behavioral alterations in mice. Notably, butyric acid-producing bacteria, particularly *g_Butyricimonas*, play a pivotal role in this process. Butyrate, a metabolite produced by gut microbiota, has been shown to modulate acute stress-induced ferroptosis of PFC neurons and associated depression-like behaviors through the gut–brain axis. This research identifies potential targets for the prevention and treatment of stress-related mental disorders mediated by gut microbiota.

## Figures and Tables

**Table 1:** Primers for RT-qPCR reactions.

Primer	Sequences (5′-3′)
GAPDH (F)	GGTGAAGGTCGGTGTGAACG
GAPDH (R)	CTCGCTCCTGGAAGATGGTG
IL-1β (F)	ATCTCGCAGCACATCA
IL-1β (R)	CCAGCAGGTTATCATCATCATCC
IL-6 (F)	CTTGGGACTGATGCTGGTGAC
IL-6 (R)	TTCTCATTTCCACGATTTCCCA
TNF-α (F)	TCAGAATGAGGCTGGATAAGAT
TNF-α (R)	GAGGAGGCAACAAGGTAGAG
IL-4 (F)	ACAGGAGAAGGGACGCCAT
IL-4 (R)	GAAGCCCTACAGACGAGCTCA
IL-10 (F)	GCCAGAGCCACATGCTCCTA
IL-10 (R)	GATAAGGCTTGGCAACCCAAGTAA

## Data Availability

The raw data supporting the conclusions of this article will be made available by the authors, without undue reservation.

## Supplementary Materials

The following supporting information can be downloaded at: https://www.mdpi.com/article/10.3390/ijms26041698/s1.
