# Supplementary material for: The Gut Microbiota Metabolite Butyrate Modulates Acute Stress-Induced Ferroptosis in the Prefrontal Cortex via the Gut–Brain Axis
Source: Int J Mol Sci. 2025 Feb 17;26(4):1698. doi: 10.3390/ijms26041698 (PMC11855447; doi:10.3390/ijms26041698)
Supplement: Supplementary file 1 [file ijms-26-01698-s001.zip › ijms-3465155-supplementary.pdf]

International Journal of
Molecular Sciences

MDPI

Supplementary Materials

The Gut Microbiota Metabolite Butyrate Modulates Acute Stress-Induced Ferroptosis in the Prefrontal Cortex via the Gut–Brain Axis

Zhen Wang [1,†], Xiaoying Ma [1,†], Weibo Shi [1], Weihao Zhu [1], Xiaowei Feng [1], Hongjian Xin [1], Yifan Zhang [1], Bin Cong [1,*] and Yingmin Li [1,*]

[1] Collaborative Innovation Center of Forensic Medical Molecular Identification, Hebei Key Laboratory of Forensic Medicine, Department of Forensic Medicine, Hebei Medical University, Shijiazhuang 050017, China; 22033100273@stu.hebmu.edu.cn (Z.W.); 22031100052@stu.hebmu.edu.cn (X.M); shiweibo@hebmu.edu.cn (W.S.); 24031100106@stu.hebmu.edu.cn (X.F.); 22033100279@stu.hebmu.edu.cn (H.X.); 23033100279@hebmu.edu.cn (Y.Z.);

* Correspondence: cong6406@hebmu.edu.cn (B.C.); 16000557@hebmu.edu.cn (Y.L.); Tel.: +86-31186261004 (Y.L.)

† These authors contributed equally to this work.

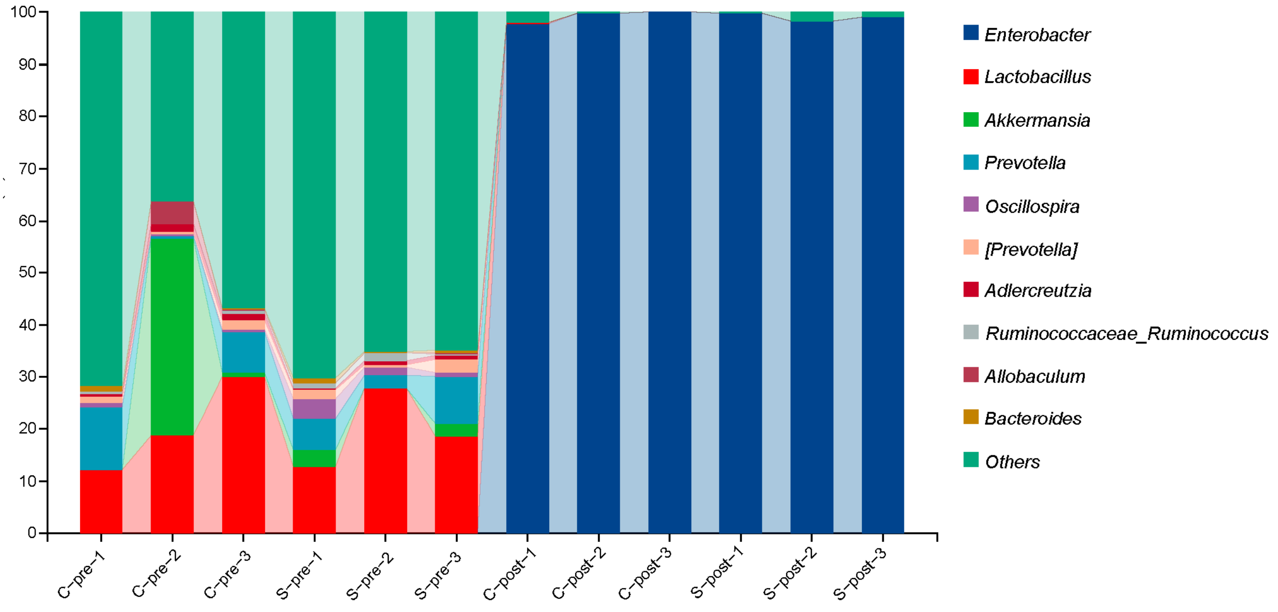

Figure S1. Relative abundance of gut microbiota at the genus level before and after antibiotic treatment (n = 6).

 https://doi.org/10.3390/ijms26041698
